# Urinary tract infections among pregnant women in rural West Amhara, Ethiopia: Prevalence, bacterial etiology, risk factors, and antimicrobial resistance patterns

**DOI:** 10.21203/rs.3.rs-5737078/v1

**Published:** 2025-01-09

**Authors:** Mulatu Melese Derebe, Unmesha Roy Paladhi, Firehiwot Workneh, Abaineh Munshea, Gizachew Yismaw, Kalkidan Yibeltal, Nebiyou Fasil, Alemayehu Worku, Tsehaynesh Gebreyesus, Wudu Tafere, Alem Tsega, Parul Christian, Rose L. Molina, Blair J. Wylie, Yemane Berhane, Anne CC Lee

**Affiliations:** Bahir Dar University; Warren Alpert Medical School, Brown University; Addis Continental Institute of Public Health; Bahir Dar University; Amhara National Regional State Public Health Institute; Addis Continental Institute of Public Health; Addis Continental Institute of Public Health; Addis Continental Institute of Public Health; Amhara National Regional State Public Health Institute; Amhara National Regional State Public Health Institute; Amhara National Regional State Public Health Institute; Teachers College, Columbia University; Beth Israel Deaconess Medical Center; Beth Israel Deaconess Medical Center; Addis Continental Institute of Public Health; Warren Alpert Medical School, Brown University

**Keywords:** UTI, Uropathogen, Pregnancy, Antimicrobial resistance, risk factor analysis

## Abstract

Urinary Tract Infections (UTIs) in pregnant women can lead to pyelonephritis and preterm birth. We assessed UTI prevalence, etiology, antimicrobial resistance, and associated risk factors among pregnant women receiving antenatal care in rural Amhara, Ethiopia. 604 pregnant women were screened for UTI at ≤ 24 weeks gestational age from August 2020 to June 2022. Urine culture, dipstick, and antibiotic sensitivity testing were completed. We conducted descriptive statistics for prevalence and logistic regression to examine UTI risk factors. UTI prevalence was 3.5% (21/604, 95%CI = 2.0%–4.9%), among which 43% were symptomatic and 57% were asymptomatic. Common uropathogens were *Escherichia coli* (57.1%), *Klebsiella pneumoniae* (14.3%), and *Enterococcus faecalis* (14.3%). Among all isolates, resistance was high for ampicillin (66.7%) and amoxicillin-clavulanate (40.0%). The majority of isolates (76.2%) were susceptible to nitrofurantoin, cotrimoxazole, and cefpodoxime. Maternal age > 20 years was a protective factor against UTI (OR = 0.27, 95% CI = 0.10–0.77; ref < 20 years). Urine dipstick (nitrite or leukocyte esterase) had low sensitivity (37.5%) but higher specificity (93.9%) to identify positive culture. This study emphasizes the high resistance to first-line antibiotics used in pregnancy and the need for accurate, low-cost UTI screening methods in LMICs.

## INTRODUCTION

Urinary tract infections (UTIs) pose a significant public health problem, with more than 150 million global annual incident cases^1^ and a global prevalence as high as 23.9% among pregnant women.^2^ Pregnant women experience UTIs at a higher rate than non-pregnant women, which may lead to a higher risk of morbidity^3^. UTIs during pregnancy range from asymptomatic bacteriuria (ASB) and acute cystitis to more serious conditions like pyelonephritis^4^. Adverse birth outcomes associated with UTIs in pregnancy include preterm birth, intrauterine fetal growth restriction, and low birth weight^5^. In pregnant women, UTIs may be associated with maternal complications including pregnancy-induced hypertension, preeclampsia, transient renal insufficiency, sepsis, anemia, and, in rare cases, death^6,7^.

The UTI prevalence of pregnant women in Ethiopia ranges between 9.5%–26.6%^8,9^, with ASB affecting 2–7% of these women^10^. It is essential for healthcare providers to effectively identify, diagnose, and treat UTIs in pregnant women to prevent adverse maternal and birth outcomes. The WHO antenatal care guidelines and Infectious Disease Society of America Clinical Practice Guidelines recommend a single-screening by urine culture and treatment approach based on antimicrobial susceptibility testing (AST)^11,12^. Implementing this strategy in low- and middle-income countries (LMICs) like Ethiopia remains challenging due to the high costs and logistical complexities associated with performing urine culture^13^.

Understanding the bacterial etiology of UTIs and antibiotic sensitivity patterns is critical to selecting the appropriate antibiotic for treatment. *Escherichia coli* is a leading cause of UTIs in Africa and Asia during pregnancy. At the same time, other bacteria such as *Klebsiella pneumoniae*, *Proteus mirabilis*, *Enterobacter species*, *Staphylococcus saprophyticus*, and group B beta-hemolytic *Streptococcus* are also common in this region. In a systematic review of antibiotic resistance patterns, most uropathogens in Asia and Africa showed high resistance to ampicillin (67.2%) with relative sensitivity to nitrofurantoin (65%), ciprofloxacin (71.2%), and ceftriaxone (74.1%)^14^. In one prior study in antenatal care clinics in Ethiopia, *E. coli* was the predominant isolated pathogen among pregnant women with the most resistance to amoxicillin (81%), followed by amoxicillin-clavulanic acid (80%). In addition, the second most significant pathogen was *Klebsiella* species, which had more resistance to ampicillin (76%), and both pathogens showed less resistance to Nitrofurantoin and ceftriaxone^15^.

Pregnancy is a critical period that increases the likelihood of UTIs due to various risk factors, such as hormonal changes, physiological changes in the urinary tract, and the growing uterus exerting pressure on the bladder. These factors increase the susceptibility of pregnant individuals to infections and underscore the importance of effective management and treatment. Therefore, it is important to determine the risk factors for UTIs in these settings to target better screening, prevention, and treatment strategies to mitigate the negative impacts of UTIs during pregnancy. Previous studies done in high-income countries and LMICs like Ethiopia have identified risk factors for UTIs, including a prior history of urinary tract infections, diabetes mellitus, high parity, low socioeconomic status, immunosuppression, gestational age, advanced maternal age, and late presentation for prenatal care^7,16,17^. Although these important foundational works have already been conducted, there is limited data from rural settings in Ethiopia where pregnancy care may look very distinct from that in urban settings in LMICs. Thus, to contribute to the body of evidence, this study aimed to assess the prevalence of UTIs, their associated risk factors, and the antimicrobial susceptibility profile of bacterial isolates among pregnant women receiving ANC services in rural areas of northwest Ethiopia.

## METHODS

### Study design and settings

The Enhancing Nutrition and Antenatal Infection Treatment (ENAT) study (ISRCN Registry: ISRCTN15116516) was conducted from August 2020 to June 2022 in the rural districts of the West Gojjam and South Gondar zones, in Amhara, Ethiopia.^18^ The ENAT study was conducted in 12 health centers selected based on accessibility, ANC patient volume, and proximity to the regional reference laboratory for sample transportation and microbiological diagnosis. Six of the study sites were randomized to receive an “Enhanced Nutrition Package” (ENP) intervention and the other six received routine nutrition care. Women in the ENP arm received iodized salt and iron-folic acid supplements, and women with a middle-upper arm circumference (MUAC) < 23 cm received a micronutrient-fortified Balanced Energy Protein (BEP). In all 12 sites, individual women were randomized to receive either the “Enhanced Infection Management Package” (EIMP) intervention or standard infection care. Women in the EIMP arm received screening and treatment for UTI/ASB and sexually transmitted infections/reproductive tract infections at enrollment, as well as presumptive deworming in the second trimester followed by stool screening and treatment at least four weeks later. Those in the standard care arm received syndromic management of diseases and health systems strengthening. The full ENAT study protocol has been published.^18^ In the current study, we use prospective data from the EIMP study arm of the parent study and examine the prevalence and associated risk factors of UTI among pregnant women within the EIMP arm.

### Study population

Pregnant women with a viable pregnancy identified at ≤ 24 weeks gestation who were enrolled into the EIMP study arm and had UTI screening were included in this analysis.

### Study procedures

After a study nurse explained the procedures to potential participants presenting for their ANC visit and obtained consent, data were collected on their background, sexual history, and nutritional intake. Women were asked to provide a urine sample for urine culture and antibiotic susceptibility testing. Urine screening by culture was performed for 604 participants from August 2020 to June 2022.

### Sample collection and processing

The study nurses and health center laboratory technicians provided participants with detailed instructions on collecting a clean-mid-catch urine sample. Job aids posted on the wall and available at the tables were used to orient the mother on collecting and transporting the sample to the lab without contamination. A sterile container was used to collect a 20–30 ml clean catch midstream urine sample, which was transferred to a boric acid-containing vacutainer for transport and storage at room temperature until processing (Beckton Dickinson Urine collection kit: BD 364956) The BD sample collection tube allowed for preserving the urine for up to 48 hours before plating at APHI. Specimens were transported to the microbiology reference laboratory of the Amhara Public Health Institute (APHI) for inoculation, culture, and incubation for 48 hours. Full biospecimen collection and processing details are described elsewhere^19^.

A randomly chosen subset of participant urine samples was also tested using a urine dipstick. These dipstick samples were visually assessed by the health center laboratory technician against a standard color chart for levels of leukocyte esterase, protein, glucose, nitrite, and ketones. The Combur10-Test M-strip was utilized following the manufacturer’s instructions (Roche) at study health centers.

### Urine culture and antimicrobial sensitivity test

At APHI, the urine specimens were inoculated onto Blood agar and MacConkey plates by using a calibrated 0.001ml (1 microliter) inoculating loop and then incubated aerobically at 37°C for 48 hrs. The growth of bacterial pathogens was inspected usually and graded for the presence of significant bacteriuria. The quantification of the colony was done by multiplying the colony count by 1000 to estimate the number of bacteria per ml of urine (CFU/ml). Urine culture demonstrated significant bacteriuria was further identified by their colony characteristics appearance. The identification and Antimicrobial Sensitivity Test *(*AST) were conducted by using the Vitek-2 compact method with the Biomet Rieux supply kits GN-71 and GP-71 for identification and AST-GN71 and AST-GP 71 for AST (RIF413 402) for both gram-negative and gram-positive bacteria respectively. Well-isolated bacterial colonies were emulsified with 3 ml of 0.45% saline. The inoculum turbidity was adjusted to match 0.5 McFarland standard solutions. For AST of Gram-negative and Gram-positive bacteria, 145 μl and 280 μl volume of suspension respectively were taken from the original suspension. Identification and antimicrobial susceptibility testing cards were loaded into the VITEK-2 Compact software to determine the bacterial species and their AST profiles. The drugs used in the susceptibility test were amoxicillin-clavulanic acid (AMC), ampicillin (AMP), cefazolin (CFZ), cotrimoxazole (COT), cefpodoxime (CPD) and nitrofurantoin (NFT).

### Definitions of outcome

High-burden bacterial growth was defined as bacteriuria of ≥ 10^5^ CFU/mL of urine of a single uropathogen and intermediate growth as bacteriuria with ≥ 10^3^ to < 10^5^ CFU/mL of a single uropathogen. Contaminated samples were defined as bacterial growth of > 2 microorganisms or growth of a non-urinary tract pathogen based on ASM standard^20^. We defined symptomatic UTI as women with ≥ 10^3^ bacteriuria with UTI signs and symptoms, including dysuria, urinary frequency, hematuria, fever, abdominal pain, flank pain, and the urgency of urination. Asymptomatic Bacteriuria (ASB) was defined as bacterial colony counts ≥ 10^5^ CFU/mL from urine samples collected from a person without UTI signs and symptoms. Any UTI was defined as both symptomatic UTI and ASB.

### Quality Assurance

The study nurses and laboratory technicians at each of the study sites were trained on urine sample collection, short-term storage, transportation, and long-term storage. The principal investigator provided intensive supervision and observed the sample collection and transportation. The SOPs of the microbiology laboratory of the APHI were followed. The sterility of the media was checked by overnight incubation at 37°C before inoculation, and Quality Control (QC) strain bacteria such as *S. aureus* (ATCC strain 25923), *P. aeruginosa* (ATCC 27853) and *E. coli* (ATCC strain 25922) were used to check the growth support of the media. The VITEK-2 software used for QC testing automatically reads the expiration date and card type information and then passes the QC run. All the data were collected on tablet computers into Survey Solutions and checked for completeness.

### Statistical analysis

We conducted basic descriptive statistics of UTI prevalence along with the corresponding 95% confidence interval, uropathogen distribution, and antibiotic sensitivity patterns. We used bivariable and multivariable binary logistic regression modeling to assess the associated risk factors. The primary outcome of every model was any UTI, as defined previously. The bivariate model included all potential risk factors as exposures, and the outcome was UTI. The exposures included in the bivariate model were chosen after conducting a literature review of factors included in the UTI risk factor analyses for which we had sufficient numbers. The following exposures were included: age, education, partner education, occupation, household size, cow/ox ownership, land ownership, drinking water source, toilet type used, parity, gestational age, MUAC, and BMI. Factors determined to be statistically significant (p ≤ 0.1) in bivariate models were included in the multivariate model (age, education, and BMI). Given the co-linearity of parity and age, only age was included in the adjusted model.

The diagnostic accuracy of the urine dipstick test (determined by nitrite and leukocyte esterase) was compared to that of urine culture, which is considered the gold standard. The accuracy of the test was assessed by calculating its sensitivity, specificity, positive predictive value (PPV), negative predictive value (NPV), and positive (+ LR) and negative (− LR) likelihood ratios. The analysis focused on nitrite positivity (NIT+), leukocyte esterase positivity (LE+), either test being positive (NIT + or LE+), and the combination of both tests being positive (NIT + and LE+) related to positive urine results. All analyses were conducted via STATA version 18.0 (StataCorp LP, College Station, TX).

### Ethics approval

The ENAT study protocol was evaluated and approved by the Institutional Review Boards of Mass General Brigham (2018P002479), USA, and the Addis Continental Institute of Public Health (001-A1-2019), Addis Ababa, Ethiopia. Ethical approval was obtained from Bahir Dar University (PRCSVD/168/213), College of Science Ethics Committee, Bahir Dar, Ethiopia. We received approval and an official support letter from the Amhara Regional State Public Health Institute. Additionally, permissions were obtained from the woredas and the 12 study health centers. A written informed consent was obtained before data collection. All methods were performed in accordance with the relevant guidelines and regulations.

## RESULTS

**Figure 1:**
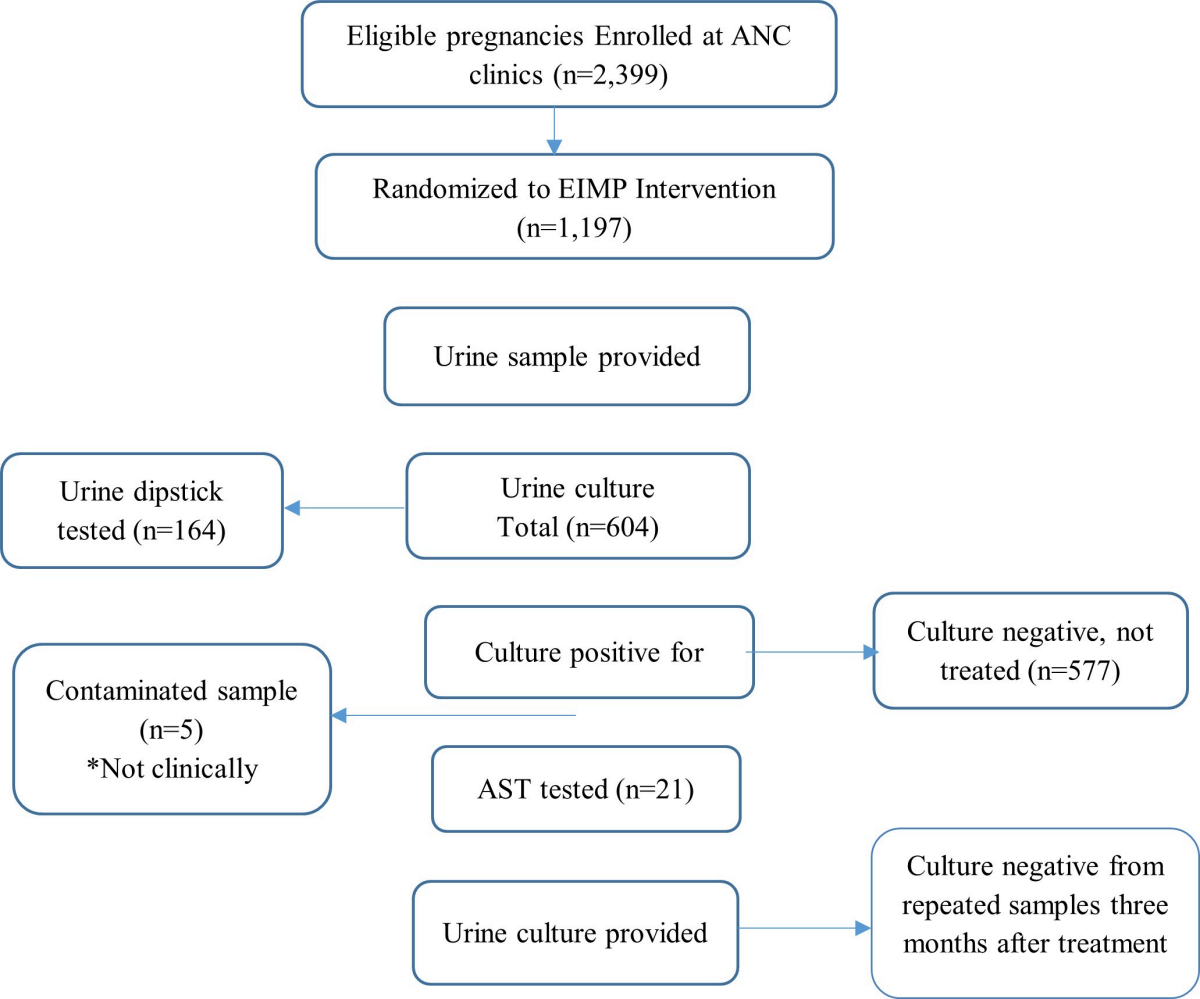
Flow Chart to patient enrollment and specimens collected as part of ENAT study intervention **ANC**: Antenatal care; EIMP: Enhanced infection management package; **UTI**: Urinary tract infection; **AST**: Antimicrobial sensitivity test. *Not clinically significant, significant growth of UTI bacteria but asymptomatic; did not fulfill criteria for UTI treatment.

Among the 2,399 women enrolled 1,197 were individually randomized to the EIMP intervention (Figure 1). A total of 604 women were screened for urine by culture during the urine screening period. Among the 604 samples, 27 (4.5%) showed bacterial growth, 577 (95.5%) had no growth, and five were contaminated. Of the 27 culture-positive samples, 21 were identified as significant uropathogens (3.5%) and further tested for antimicrobial sensitivity test (AST).

*Contaminated samples,* samples of bacterial growth of >2 microorganisms or growth of non-urinary tract pathogens.

### Prevalence of UTIs

Among those included in this study, 3.6% (95% confidence interval [CI]= 2.2%–5.2%), (22/604) of cultures had significant or high-burden bacterial growth (Table 1). Asymptomatic bacteriuria (ASB) accounted for 59.1% (13/22) of cases, and 40.9% (9/22) were symptomatic (Table 1). The 21 women were treated with selected antibiotics based on antimicrobial resistance patterns, and follow-up testing confirmed that all treated patients were cured. However, of the 22 participants exhibiting growth, one case was excluded as she didn’t fulfill clinical significance criteria as UTI pathogens and treatment among ASB.

### Sociodemographic characteristics and UTIs

In this study, the majority of women (89.6%) were greater than or equal to the age of 20, with a mean age of 26.3 years (standard deviation [SD]: 5.7). Most participants (97.0%) were married, and a large proportion (96.3%) self-identified as Orthodox Christians. Nearly half (46.2%) had no formal education, and 47.4% worked in agriculture, farming, or as daily laborers. Almost three-quarters (71.3%) of the women were multiparous (Table 2).

### Risk factors for UTIs

We evaluated 13 independent factors via bivariate analysis to identify risk factors of UTI. In the bivariate analysis, higher maternal age ≥20 (Odds Ratio [OR]=0.27, 95% CI=0.10–0.72), and higher parity (OR=0.43, 95% CI=0.18–1.03) were associated with lower UTI risk. There was a borderline association between maternal underweight and UTI, although this did not reach statistical significance (Table 3).

The risk factors with p-value ≤0.1 in the bivariate model were included in the multivariable analysis: women’s age at enrollment, education, parity, and undernutrition at enrollment (BMI<18.5). In this multivariable analysis, only older maternal age (≥20 years) was associated with lower UTI risk (OR=0.27, 95% CI=0.10–0.77, p=0.014).

### Etiology of UTI

**Figure 2:**
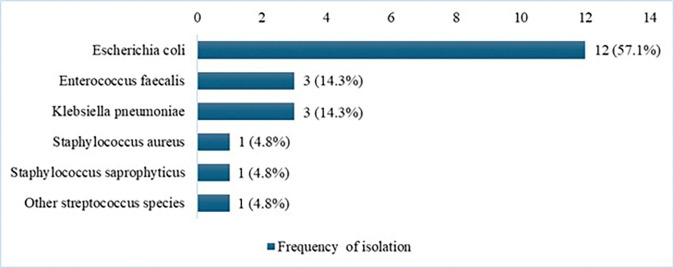
Etiology of uropathogens isolated from pregnant women diagnosed with UTI in rural Amhara, Ethiopia, 2020–2022.

Figure 2 presents the bacterial causes of UTIs identified from urine culture tests among the 21 women who showed either high-burden growth or significant growth and were symptomatic. Six bacterial species were identified. *E. coli* was the most prevalent, accounting for 57.1% (12/21) of the total isolated uropathogens. *Klebsiella pneumoniae* (14.3%, 3/21), *Enterococcus faecalis* (14.3%, 3/21), *Staphylococcus aureus* (4.8%, 1/21), *Staphylococcus saprophyticus* (4.8%, 1/21), and other *Streptococcus species* (4.8%, 1/21) were the remaining cases. *E. coli* was isolated more commonly, with 58.3% (7/12) of women exhibiting UTI symptoms. The remaining isolates tended to present more commonly as asymptomatic bacteriuria: *Klebsiella pneumoniae* (66.7%, 2/3), *Enterococcus faecalis* (100%, 3/3), *Staphylococcus saprophyticus* (100%, 1/1), and other *Streptococcus species* (100%, 1/1) were all isolated from asymptomatic UTI patients (Supplementary Table S1).

### Antimicrobial susceptibility patterns

Table 4 and Supplementary Fig.S1 show the percentages of bacterial isolates that were classified as susceptible, intermediate, or resistant to various antibiotics. Among all of the identified uropathogens, overall rates of antibiotic resistance were high for ampicillin (66.7%, 14/21) and ampicillin-clavulanic acid (40.0%, 6/15) for gram-negative uropathogens. The highest overall rates of antibiotic susceptibility were for cotrimoxazole, cefpodoxime, and nitrofurantoin (each 76.2%, 16/21); followed by cefazolin (61.9%, 13/21); and much lower for ampicillin (28.6%, 6/21). Conversely, by species, *E. coli* had the highest resistance to ampicillin (66.7%, 8/12) and amoxicillin-clavulanic acid (50.0%, 6/12), *Enterococcus faecalis* exhibited the highest resistance to ampicillin (66.7%, 2/3) but was fully susceptible to cefpodoxime and nitrofurantoin (100%, 3/3). Furthermore, all the *Klebsiella pneumoniae* isolates were resistant to ampicillin (100%, 3/3) but were fully susceptible to cefpodoxime (100%, 3/3).

### Urine dipstick results with diagnostic accuracy compared with culture results

The accuracy of the dipstick test for nitrite and leukocyte esterase was assessed by comparing its sensitivity and specificity to those of culture, which is the gold standard. Nitrite testing (NIT+) alone exhibited a relatively low sensitivity of 28.6% (95% CI=3.7–70.9) but had a high specificity of 98.6% (95% CI=95.2–99.8). The positive predictive value (PPV) for nitrite was 50.0% (95% CI=14.1–85.9), and the negative predictive value (NPV) was 96.7% (95% CI=94.8–97.9). Leukocyte esterase (LE+) testing alone had a lower sensitivity of 14.3% (95% CI=0.4–57.9) and a high specificity of 96.2% (95% CI=91.9–98.6), with a PPV of 14.3% (95% CI=2.3–54.6). When nitrite-positive or leukocyte esterase-positive results were combined, the sensitivity and specificity were 37.5% (95% CI=8.5–75.5) and 93.9% (95% CI=88.2–97.3), respectively. Additionally, compared with culture results, UTI symptoms had a sensitivity of 42.9% (95% CI=1.7 – 14.8) and specificity of 64.2% (95% CI=91.8 – 99.1) (Table 5).

## DISCUSSION

The prevalence of UTI, including both symptomatic and asymptomatic bacteriuria, was 3.5% among pregnant women in rural Amhara. The majority of UTIs were asymptomatic; however, the commonly used urine dipstick was an inadequate screening tool for detecting UTIs, given the low sensitivity. *E. coli* was the most common uropathogen, followed by Klebsiella and Enterococcus. The majority of isolates were susceptible to nitrofurantoin, cotrimoxazole, and cefpodoxime, while resistance was high for ampicillin. This has relevant implications given that amoxicillin is the current first-line treatment for UTI in pregnant women in Ethiopia.

Several studies across Ethiopia indicate that the prevalence of UTIs among pregnant women ranges from 9% to 26.6%^16^. Our study, conducted in rural Amhara, Ethiopia, reported a much lower prevalence of 3.5%, which is consistent with findings from Pakistan, India, Turkey, and Bangladesh^21–24^. In contrast, higher prevalence rates (9%–30%) have been observed in various LMICs across Asia and Africa^16,25–28^. The lower prevalence in our study may be due to differences in screening populations as our population was a very rural low-risk patient population in outpatient ANC compared to other settings that may have recruited more urban or high-risk populations, patient demographics, socioeconomic factors, hygiene practices, access to ANC service, and urine collection methods^29^. We also did extensive training of laboratory staff and patients to collect urine specimens using aseptic techniques and rates of urine contamination and growth were lower than other studies. Furthermore, a recent systematic review and meta-analysis in Ethiopia also identified the Amhara region as having the lowest UTI burden (9.8%)^16^. The most common pathogen isolated was *E. coli* (57.1%), consistent with studies from Asia and Ethiopia^30–34^. *Klebsiella pneumoniae and Enterococcus faecalis* (14.3%) were the second most common isolated pathogens, aligning with findings from Nigeria and Nepal^31,35^.

The common risk factors identified in previous studies included socio-demographic characteristics (age, education, marital status, occupation, family income ) and other clinical factors such as history of UTI, parity, gravidity, gestational age, and presence of other infections linked with UTIs^36^. Though maternal age, education, parity, and undernutrition (BMI <18.5) were statistically significant or of borderline significance in univariate analysis, only maternal age remained significant in multivariable analysis, which aligns with findings from Eastern Ethiopia^37^. This suggests that targeting screening with urine culture based on risk factors may not be the optimal approach to identifying UTI cases in pregnant women in similar settings and populations. Other factors examined here, such as education, occupation, household size, cow/ox ownership, land ownership, drinking water source, toilet type used, parity, gestational age, and undernutrition with MUAC and BMI were not statistically significant and findings aligned with studies from Sudan and Tanzania^26,38^, and other regions of Ethiopia^36,39–41^. In this study, differences for certain known risk factors, such as undernutrition (BMI), gestational age, and marital status, may not have been observed due to the small sample size and number of UTI cases across the comparison groups. Parity was excluded from the multivariable analysis because of its collinearity with maternal age. Additionally, UTI screening was only done at a one-time point at enrollment <24 weeks of gestation, we may have systematically excluded women who could exhibit different UTI risks later in pregnancy.

Antimicrobial resistance among uropathogens is a critical challenge for managing UTIs, particularly in LMICs, where there is more limited antibiotic availability for UTIs. This study showed high resistance rates to commonly prescribed antibiotics for UTIs in pregnancy in Ethiopia, such as ampicillin/amoxicillin (66.7%), which is the first-line treatment for dysuria in primary care settings. Similarly, nearly half of the isolates were resistant to amoxicillin-clavulanic acid, aligning with studies from Addis Ababa and Dessie, Ethiopia^42,43^. *E. coli* also exhibited significant resistance to ampicillin, similar to Nigerian and Ethiopian studies^35,37,42,44,45^, and *Klebsiella pneumoniae*, the second most common uropathogen, was fully resistant to ampicillin. While susceptibility was higher to antibiotics like nitrofurantoin and cefpodoxime, both antibiotics were not routinely available in health centers and difficult to procure in the study catchment area. On a few occasions for the study, a supply was shipped from Addis Ababa to study sites for study participant treatment. This highlights the problem of effectively treating UTIs in primary care settings in Amhara. These findings underscore the urgent need for understanding antibiotic susceptibility patterns in the specific region and context and improving antibiotic access to medications safe in pregnancy to effectively treat UTIs during pregnancy, such as nitrofurantoin, in resource-limited settings.

In low-resource settings, routine screening of all pregnant women by urine culture is often not feasible given resources, laboratory capacity, and cost. UTI is typically identified by maternal symptoms or urine dipstick tests in resource-limited settings. Effective UTI screening requires high sensitivity to identify cases needing treatment. We found that dipstick tests for nitrites and leukocyte esterase both individually, and in combination, had very poor sensitivity despite high specificity, consistent with findings from Kenya, Ghana, and Ethiopia^46–48^. While combined dipstick results (“NIT+ or LE+”) had higher sensitivity, they still did not pick up almost two out of three UTIs. Symptom-based identification of UTIs also showed poor sensitivity only identifying about half of UTIs. Therefore, our results corroborate the limited value of a symptom or dipstick-based approach to UTI diagnosis, reiterating previous findings^48–50^. The difficulty of conducting urine cultures in similar settings highlights the urgent need for more accessible and accurate rapid and low-cost diagnostic tools to improve UTI management and maternal health outcomes.

To our knowledge, this study is one of the first population-based studies on UTI in pregnant women from rural health centers in the Amhara region. The use of the BACTEC-2 system for pathogen identification and antimicrobial susceptibility testing is a notable strength, offering better sensitivity and minimizing technical errors than traditional methods such as morphological identification and disk diffusion AST. However, the study had limitations. It only included UTI screening of pregnant women ≤24 weeks gestation, and did not evaluate UTI prevalence or risks in later pregnancy. The relatively small number of UTI cases may have reduced the study’s power to detect differences between the study groups. Lastly, the lack of availability of certain antibiotic AST testing, such as azithromycin and ceftriaxone, restricted the assessment of AST patterns for all potential treatment options in the primary care settings of the study areas.

## CONCLUSION

In this study, the prevalence of UTIs among pregnant women in rural Northwest Ethiopia is lower than other previous studies in Ethiopia. Isolated pathogens were resistant to the common first line antimicrobials used in pregnancy, with important implications for treatment options of UTIs among pregnant women in this setting. *E. coli* was the predominant isolate, with high resistance to amoxicillin-clavulanic acid and ampicillin. Maternal age under 20 was a significant risk factor. Urine dipstick tests showed limited diagnostic accuracy and poor sensitivity compared to culture but may help rule out UTIs when negative. Given the limitations of universal urine culture in LMICs and the dipstick’s poor accuracy, improved point-of-care diagnostics are urgently needed. This study underscores the need for expanded antibiotic access, better diagnostics, and further research through larger, inclusive studies to address antibiotic resistance, optimize diagnostic tools, and enhance UTI management in rural LMIC settings.

## Figures and Tables

**Table 1.** Prevalence of UTIs among pregnant women receiving ANC in the rural population of West Amhara, Ethiopia, 2020–2022.

Burden of bacterial growth	Total (N=604) n (%)	Symptomatic UTI* (N=9) n	Asymptomatic UTI* (N=13) n
No growth	577 (95.5)	-	-
High burden growth (>=100,000 CFU/ml)	20 (3.3)	8.0	12.0
Significant growth (10,000–99,999 CFU/ml)	2 (0.3)	1.0	1.0
Contaminated sample**	5 (0.8)	-	-
Total	604	9.0	13.0

* Symptomatic UTIs were defined as women with chief complaints of dysuria, urinary frequency, hematuria, abdominal pain, fever, or flank pain.

* Asymptomatic UTIs were defined as women with significant or high-burden bacterial growth without UTI symptoms.

** Samples were considered contaminated when there was bacterial growth of >2 microorganisms or growth of non-urinary tract pathogens.

**Table 2.** Characteristics of pregnant women attending ANC Visits in the rural population of West Amhara, Ethiopia, 2020–2022.

Variables	No UTI (N=583) n (%)	UTI^§^ (N=21) n (%)	Overall (N=604) n (%)

**Age at enrollment**			
< 20	57 (9.8)	6 (28.6)	63 (10.4)
≥ 20	526 (90.2)	15 (71.4)	541 (89.6)

**Marital status**			
Married	564 (97.2)	19 (90.5)	583 (97.0)
Divorced/separated or never married	16 (2.8)	2 (9.5)	18 (3.0)

**Religion**			
Orthodox Christian	558 (96.2)	21 (100.0)	579 (96.3)
Muslim	22 (3.8)	0 (0.0)	22 (3.7)

**Education**			
No education	271 (46.7)	7 (33.3)	278 (46.3)
Primary	175 (30.2)	6 (28.6)	181 (30.1)
Secondary and above	134 (23.1)	8 (38.1)	142 (23.6)

**Partner Education**			
No education	259 (44.7)	7 (33.3)	266 (44.3)
Primary	178 (30.7)	9 (42.9)	187 (31.2)
Secondary and above	142 (24.5)	5 (23.8)	147 (24.5)

**Occupation**			
Housewife	184 (31.7)	8 (38.1)	192 (32.0)
Wage occupation	121 (20.9)	3 (14.3)	124 (20.6)
Agriculture/farmer/daily laborer	275 (47.4)	10 (47.6)	285 (47.4)

**Household size**			
1–2 people	196 (33.6)	10 (47.6)	206 (34.1)
≥3 people	387 (66.4)	11 (52.4)	398 (65.9)

**Cow/ox ownership**			
No	215 (37.1)	10 (47.6)	225 (37.4)
Yes	365 (62.9)	11 (52.4)	376 (62.6)

**Land ownership**			
No	182 (31.4)	5 (23.8)	187 (31.1)
Yes	398 (68.6)	16 (76.2)	414 (68.9)

**Drinking water source**			
Public tap	364 (62.8)	10 (47.6)	374 (62.2)
Other water sources (spring, surface & other)	216 (37.2)	11 (52.4)	227 (37.8)

**Toilet type used**			
No toilet	197 (34.0)	5 (23.8)	202 (33.6)
Any latrine/toilet	383 (66.0)	16 (76.2)	399 (66.4)

**Parity** *			
Primiparous	163 (28.1)	10 (47.6)	173 (28.7)
Multiparous	418 (71.9)	11 (52.4)	429 (71.3)

**Gestational age at urine collection** **			
1–12 weeks	112 (19.2)	6 (28.6)	118 (19.5)
13–24 weeks	471 (80.8)	15 (71.4)	486 (80.5)	

**Undernutrition (MUAC < 23)**			
Yes (<23 cm)	178 (31.3)	9 (42.9)	187 (31.8)	
No (>23 cm)	390 (68.7)	12 (57.1)	402 (68.2)	

**Undernutrition (BMI)**			
Underweight (18.5)	84 (14.4)	6 (28.6)	90 (14.9)	
Normal weight (18.5–24.9)	465 (79.8)	15 (71.4)	480 (79.5)	
Overweight/obese (>=25)	34 (5.8)	0 (0.0)	34 (5.6)	

§ UTI was defined as an infection that affects a part of the urinary system, which includes the kidneys, bladder, ureters, and urethra.

* Parity is the number of pregnancies carried out by a female for at least 20 weeks.

** Gestational age is the age of a pregnancy taken from the beginning of the woman’s last menstrual period. Missingness: 3, marital status; 3, religion; 3, education; 4, partner education; 3, occupation; 3, household size; 3, cow ownership; 3, land ownership; 3, drinking water source; 3, toilet type used; 2, parity; 15, MUAC <23; 6, BMI.

**Table 3.** Univariate and multivariate risk factor analysis of pregnant women attending ANC visits in the rural population of West Amhara, Ethiopia, 2020-2022.

Variables	Crude/Bivariable model		Adjusted/Multivariable Model	

	OR (95% CI)	p-value	aOR (95% CI)	p-value

**Age**				
< 20	Ref	-	Ref	-
≥ 20	0.27 (0.10–0.72)	0.009	0.27 (0.10–0.77)	0.014

**Education**				
No education	Ref.	-	-	-
Primary	1.33 (0.44–4.02)	0.616	1.06 (0.33–3.33)	0.924
Secondary and above	2.31 (0.82–6.51)	0.113	2.22 (0.77–6.36)	0.136

**Partner Education**				
No education	Ref.	-	_	_
Primary	1.87 (0.68–5.11)	0.222		
Secondary and above	1.30 (0.40–4.18)	0.656		

**Occupation**				
Housewife	Ref.	-		
Wage occupation	0.57 (0.15–2.19)	0.414	-	-
Agriculture/farmer/daily labor	0.84 (0.32–2.16)	0.712		

**Household size**				
1–2 people	Ref.	-		
≥3 people	0.55 (0.23–1.33)	0.189	-	-

**Cow/ox ownership**				
Yes	0.65 (0.27–1.55)	0.330	-	-
No	Ref.	-		

**Land ownership**				
Yes	1.46 (0.53–4.06)	0.464	-	-
No	Ref.	-		

**Drinking water source**				
Public tap	Ref.			
Other water sources (spring, surface, and others)	0.54 (0.23–1.29)	0.166	-	-

**Toilet type used**				
No toilet	Ref.	-		
Any latrine/toilet	0.61 (0.22–1.68)	0.338	-	-

**Parity**				
Primiparous	Ref.	-		
Multiparous	0.43 (0.18–1.03)	0.058	0.48 (0.19–1.25)	0.136

**Gestational age at the time of urine collection**				
1–12 weeks	Ref.	-	-	-
≥13–24 weeks	0.59 (0.23–1.57)	0.293		

**Undernutrition (MUAC < 23)**				
No (>23 cm)	Ref.	-		
Yes (≤23cm)	1.64 (0.68–3.97)	0.27		

**Undernutrition (BMI)**				
Normal weight (18.5–24.9)	Ref.	-	-	-
Underweight (18.5)	2.21 (0.84–5.87)	0.11	0.45 (0.17–1.21)	0.116

* Bivariate model includes age, education, parity, and undernutrition at enrollment (BMI<18.5). Abbreviations: OR, odds ratio; aOR, adjusted odds ratio; CI, confidence interval; MUAC, middle- and upper-arm circumference; BMI, body mass index; Ref, reference.

**Table 4.** Antimicrobial Susceptibility Patterns of Uropathogens in Urine Culture among Pregnant Women Attending ANC in Rural Amhara, Ethiopia (2020–2022).

Bacterial isolate	Total	S/I/R*	Antibiotics					
			Amoxicillin-Clavulanic-acid n (%)	Ampicillin n (%)	Cefazolin n (%)	Cotrimoxazole n (%)	Cefpodoxime n (%)	Nitrofurantoin n (%)
*Escherichia coli*	12	S	5 (41.7)	3 (25.0)	6 (50.0)	9 (75.0)	7 (58.3)	9 (75.0)
		I	1 (8.3)	1 (8.3)	2 (16.7)	0 (0.0)	0 (0.0)	3 (25.0)
		R	6 (50.0)	8 (66.7)	4 (33.3)	3 (25.0)	5 (41.7)	0 (0.0)
*Enterococcus faecalis*	3	S	0 (0.0)	1 (33.3)	2 (66.7)	2 (66.7)	3 (100)	3 (100)
		I	0 (0.0)	0 (0.0)	0 (0.0)	0 (0.0)	0 (0.0)	0 (0.0)
		R	0 (0.0)	2 (66.7)	1 (33.3)	1 (33.3)	0 (0.0)	0 (0.0)
*Klebsiella pneumoniae*	3	S	3 (100)	0 (0.0)	2 (66.7)	2 (66.3)	3 (100)	2 (66.7)
		I	0 (0.0)	0 (0.0)	0 (0.0)	0 (0.0)	0 (0.0)	0 (0.0)
		R	0 (0.0)	3 (100)	1 (33.3)	1 (33.7)	0 (0.0)	1 (33.3)
*Staphylococcus aureus*	1	S	0 (0.0)	0 (0.0)	1 (100)	1 (100)	1 (100)	1 (100)
		I	0 (0.0)	0 (0.0)	0 (0.0)	0 (0.0)	0 (0.0)	0 (0.0)
		R	0 (0.0)	1 (100)	0 (0.0)	0 (0.0)	0 (0.0)	0 (0.0)
*Staphylococcus saprophyticus*	1	S	0 (0.0)	1 (100)	1 (100)	1 (100)	1 (100)	1 (100)
		I	0 (0.0)	0 (0.0)	0 (0.0)	0 (0.0)	0 (0.0)	0 (0.0)
		R	0 (0.0)	0 (0.0)	0 (0.0)	0 (0.0)	0 (0.0)	0 (0.0)
*Other streptococcus species*	1	S	0 (0.0)	1 (100)	1 (100)	1 (100)	1 (100)	0 (0.0)
		I	0 (0.0)	0 (0.0)	0 (0.0)	0 (0.0)	0 (0.0)	0 (0.0)
		R	0 (0.0)	0 (0.0)	0 (0.0)	0 (0.0)	0 (0.0)	1 (100)
*Total*	21	S	8 (53.3)	6 (28.6)	13 (61.9)	16 (76.2)	16 (76.2)	16 (76.2)
		I	1 (6.7)	1 (4.8)	2 (9.5)	0 (0.0)	0 (0.0)	3 (14.3)
		R	6 (40.0)	14 (66.7)	6 (28.6)	5 (23.8)	5 (23.8)	2 (9.5)

* S/I/R; S, sensitive; I, intermediate; R, resistant

**Table 5.** Diagnostic accuracy of urine dipstick (nitrite and leukocyte esterase positive) to classify UTI (defined by positive urine culture) among pregnant women attending ANC in rural Amhara, Ethiopia (2020–2022).

Culture	Total tested N	Sensitivity % (95% CI)	Specificity % (95% CI)	PPV % (95% CI)	NPV % (95% CI)	+LR (95% CI)	−LR (95% CI)
NIT+	155	28.6 (3.7–70.9)	98.6 (95.2–99.8)	50.0 (14.1–85.9)	96.7 (94.8–97.9)	21.1 (3.5–128.8)	0.7 (0.4–1.2)
LE+	164	14.3 (0.4–57.9)	96.2 (91.9–98.6)	14.3 (2.3–54.6)	96.2 (94.9–97.1)	3.7 (0.5–27.0)	0.9 (0.7–1.2)
NIT+ or LE+	138	37.5 (8.5– 75.5)	93.9 (88.2–97.3)	27.3 (10.9–53.4)	96.1 (93.4–97.7)	6.1 (2.0–18.7)	0.7 (0.4–1.1)
UTI symptoms (dysuria, incontinence, or urgency)	604	42.9 (21.8–66.0)	64.2 (60.1–68.1)	4.1 (2.5–6.7)	96.9 (95.59–7.8)	1.2 (0.7–2.0)	0.9 (0.6–1.3)
UTI symptoms (Syndromic)/NIT/LE	187	50.0 (15.7–84.3)	65.4 (57.9–72.3)	6.1 (3.0–11.7)	96.7 (93.5–98.3)	1.4 (0.7–3.0)	0.8 (0.4–1.5)

Abbreviations: LE = leukocyte esterase; NIT = nitrite; UTI = urinary tract infection; PPV= positive predictive value; NPV= negative predictive value; −LR = negative likelihood ratio; +LR = positive likelihood ratio.

## Data Availability

All data produced or analyzed during this study are provided within this published article and supplementary file.
